# Multiple functions of volatiles in flowers and leaves of *Elsholtzia rugulosa* (Lamiaceae) from southwestern China

**DOI:** 10.1038/srep27616

**Published:** 2016-06-09

**Authors:** Feng-Ping Zhang, Qiu-Yun Yang, Gang Wang, Shi-Bao Zhang

**Affiliations:** 1 Key Laboratory of Economic Plants and Biotechnology, Kunming Institute of Botany, Chinese Academy of Sciences, Kunming 650201, Yunnan, China; 2 Yunnan Key Laboratory for Research and Development of Wild Plant Resources, Kunming Institute of Botany, Chinese Academy of Sciences, Kunming 650201, Yunnan, China; 3 Key Laboratory of Tropical Forest Ecology, Xishuangbanna Tropical Botanical Garden, Chinese Academy of Sciences, Mengla, 666303, Yunnan, China

## Abstract

Although the roles of volatile compounds have been examined separately in plant–herbivore or plant–pollinator interactions, few studies have focused on how plant scents can attract effective pollinators, repel ineffective pollinators, and defend against attacks by insect herbivores. We explored the functional significance of volatile compounds that impart a strong odor to *Elsholtzia rugulosa*, a shrub species in southwestern China. We monitored the pollinating honey bee *Apis cerana*, as well as two occasional visitors – *Vespa velutina* and a *Bombus* sp. – and an herbivorous beetle *Oxycetonia jocunda*. Behavior experiments using Y-tubes showed that honey bees were attracted primarily by floral scent while hornets and bumble bees were repelled by both the flowers and leaves. Analysis via gas chromatography-mass spectrometry revealed that these tissue types differed in their compositions and relative amounts of volatile compounds. When the plants were damaged, the average relative amounts of Elsholtzia ketone rapidly increased in the flowers and leaves. Furthermore, herbivorous beetles were strongly repelled by damaged tissues, suggesting a potential defense signaling function by these compounds. Our findings again demonstrate that scents have multiple functions in the interactions among plants and insects.

Some flowering plants bring pollinators into contact with reproductive structures by providing olfactory or visual signals, and by rewarding visitors with nectar, pollen, and oils^1^. In turn, pollinator preference and abundance influence a plant’s reproductive success^2^,^3^,^4^. Studies of floral characteristics have traditionally focused on visual signals assumed to be attractive to pollinators, including floral display size, flower color and shape, and nectar coloration^5^,^6^,^7^,^8^,^9^,^10^. However, it is possible that plant scents are also important in helping to attract pollinators and to maximize reproductive output^11^,^12^,^13^.

Volatile compounds help plants communicate and interact with their surrounding environment. These volatiles, released from leaves, flowers, and fruits into the atmosphere and from roots into the soil, provide a reproductive advantage by attracting pollinators and seed dispersers^12^,^13^,^14^,^15^. For example, scent can serve as a “private channel” to lure a specific pollinator species^14^. Some researches have reported that floral scent functions as an important attractant in the specialized pollination system of wasps within the genus *Hemipepesis* (Hymenoptera)^12^,^13^. As in many other plant–pollinator interactions, scents play a key role in pollinating seed-consuming mutualism^16^. Although much focus has been placed on their attractiveness, volatile compounds might also act as deterrents. For example, a plant usually releases small quantities of volatiles, but those relative proportions might change when that plant is attacked by herbivorous insects. An increase in the levels or types of volatiles that are emitted under such conditions suggests that these substances have a role in plant defenses^17^,^18^,^19^,^20^.

Plants of *Elsholtzia rugulosa* have a strong odor and are medicinally important in China^21^. Preliminary observations of this species have shown that its flowers are effectively pollinated by Asian honey bees (*Apis cerana*) and seed production increases in parallel with the number of pollinator visits to attain higher yields. Field observations have shown that hornets (*Vespa velutina*) and bumble bees (*Bombus* sp.) are also occasional floral visitors to these flowers (unpublished data). We visually confirmed that their heads and bodies did not make effective contact with the anthers and stigmas during visiting flowers occasionally, the longer tongues of hornets and bumble bees were unsuitable for pollination of *E*. *rugulosa*. At the study site, the few hornets and bumblebees caught in the mist net were lacking *E*. *rugulosa* pollen. No reports have been made on either the constitution of the unusual sharp odor in leaves and flowers of *E*. *rugulosa* or the significance of its function. However, whole-plant studies have examined the chemical components of its essential oils^22^. Here, we addressed the following: 1) do levels of these volatile compounds differ between flowers and leaves, 2) are some specific pollinators attracted by the scent of flowers and/or leaves, 3) can this strong odor deter ineffective pollinators, and 4) do the volatiles emitted by damaged tissues act as part of a defense mechanism against insect herbivores?

## Results

### Responses of three floral visitors to flower and leaf scents

**Figure 1:**
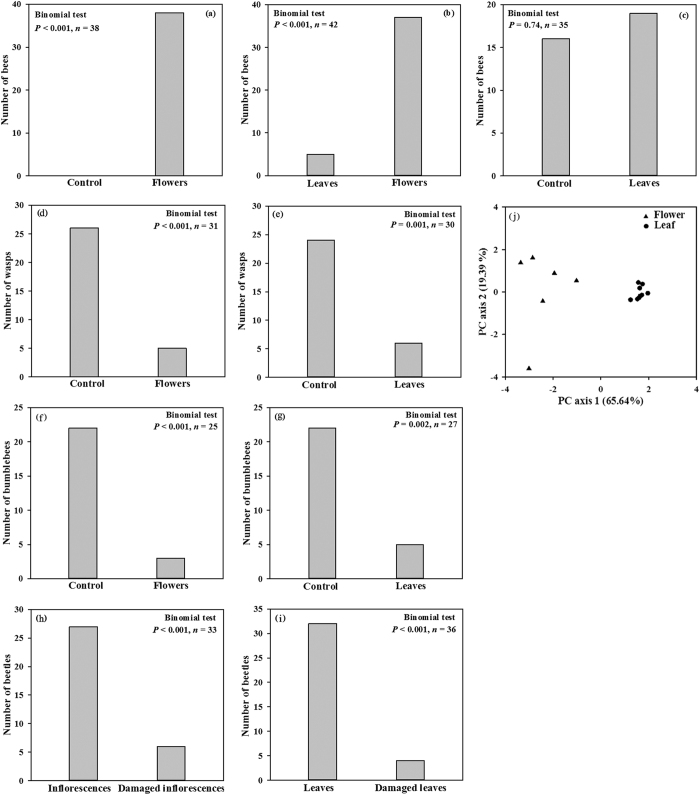
Number of *Apis cerana* honey bees choosing Y-tube branches containing *Elsholtzia rugulosa* flowers (**a**), amount visiting leaves relative to flowers (**b**) and leaves (**c**) versus preference for neither (control); Number of *Vespa velutina* hornets choosing Y-tube branches containing *Elsholtzia rugulosa* flowers (**d**) or leaves (**e**) versus preference for neither (control); Number of *Bombus* sp. bumblebees choosing Y-tube branches containing *Elsholtzia rugulosa* flowers (**f**) or leaves (**g**) versus preference for neither (control); Number of *Oxycetonia jocunda* beetles choosing Y-tube branches containing *Elsholtzia rugulosa* intact inflorescences versus damaged inflorescences (**h**) or intact leaves versus damaged leaves (**i**); Principal component (PC) analysis based on occurrence and relative amounts of volatile compounds emitted by *Elsholtzia rugulosa* flowers and leaves (**j**).

In the Y-tube experiments, *Apis cerana* bees significantly preferred the branch containing *Elsholtzia rugulosa* flowers over the empty control branch (Binomial test: *P* < 0.001, *n* = 38) (Fig. 1a). They also significantly favored the branch with flowers rather than the one with the leaves (Binomial test: *P* < 0.001, *n* = 42) (Fig. 1b). However, when offered the choice of leaves vs. empty control, neither branch was more attractive than the other (Binomial test: *P* *=* 0.74, *n* = 35) (Fig. 1c).

By contrast, the hornets and bumblebees were strongly repelled by the branches containing flowers or leaves over the empty control branch (Fig. 1d–g), and they showed an adverse reaction to the branches filled with either tissue type. Similarly, beetles strongly rejected branches containing damaged inflorescences or leaves versus branches with intact (undamaged) tissues (Fig. 1h,i).

### Analysis of flower and leaf scent compounds

The compositions and relative amounts of volatile compounds differed between flower and leaf samples (Table 1). In total, seven compounds were identified from the flowers, with the main emitted volatiles being Elsholtzia ketone (relative amount: 9.98 ± 1.26) and β-Caryophyllene (45.89 ± 2.59). By comparison, the leaf samples released only three compounds: Elsholtzia ketone (24.76 ± 4.96), Piperitone (1.59 ± 0.37), and β-Caryophyllene (3.28 ± 1.50). We found it interesting that damaged tissues emitted relatively more Elsholtzia ketone (flowers, 25.87 ± 5.93; leaves, 79.35 ± 8.07) when compared with relative levels from intact tissues (flowers, 9.98 ± 1.26, *P* = 0.05; leaves, 24.76 ± 4.96, *P* = 0.02). Principal component analysis demonstrated that the first two components explained 65.64% and 19.39% of the total variation (Fig. 1j), with axis 1 loaded by leaf on the positive side and flower on the negative side.

## Discussion

The fitness of flowering plants with specialized floral traits for pollinator attraction can decline if those flowers are also visited by animals that are ineffective pollinators^23^. This can be avoided if those traits filter out less desirable visitors while allowing suitable pollinators access to floral rewards such as nectar. For example, long corolla tubes can prevent access to all but long-tongued moths^24^,^25^. In the absence of specialized floral morphologies, however, it is sometimes unclear how plants avoid ineffective pollinators while still attracting their specialized pollinators. Some researchers have found that nectar can be unpalatable to ineffective pollinators, thereby serving as a potential floral filter^8^,^26^,^27^.

However, another mechanism not previously investigated is that plant scents are both filters and a defense against insect herbivory. This might initially seem paradoxical because their primary function is to attract animal pollinators. However, our results appear to resolve this by showing that effective pollinators find the strong odor appealing while infective pollinators are repelled. Because we also suspect that plant volatiles provide a means to defend against insect herbivores, we suggest that these volatiles from *E*. *rugulosa* play multiple roles.

Results from our GC-MS analysis showed that flowers and leaves varied in their composition and relative amounts of volatile compounds, with flowers releasing primarily Elsholtzia ketone and β-Caryophyllene and leaf emissions being dominated by Elsholtzia ketone. It was found that the damaged tissues emitted relatively more Elsholtzia ketone when compared with relative levels from intact tissues. Moreover, beetles strongly rejected branches containing damaged inflorescences or leaves versus intact tissues in Y-tube experiments. The combination of GC-MS results with behavioral experiments exhibited that Elsholtzia ketone appears to mediate the repel function, while β-Caryophyllene may play an important role in pollinator attraction. β-Caryophyllene is considered as one of the most widespread sesquiterpene floral volatiles, which occurs in floral odors in more than 50% of angiosperm families, and is one of the 12 most common volatile compounds in floral scents^28^, and it has been reported to play an important attraction role^29^. Moreover, the damaged flowers emitted more Elsholtzia ketone (9.98 vs. 25.87), while β-Caryophyllene changed little (45 vs. 40.24, Table 1). The results supported a role for Elsholtzia ketone in repulsion, while the main function of β-Caryophyllene may attract the pollinator.The seven compounds were identified from the flowers, By comparison, the leaf samples released only three compounds, the function of the 4 extra compounds of flowers may play roles in antibacterial, antifungal and attraction^30^,^31^,^32^.

Principal component analysis demonstrated that the first two components explained 65.64% and 19.39% of the total variation (Fig. 1j), with axis 1 loaded by leaf on the positive side and flower on the negative side. This allowed us to classify those tissues into distinct groups of attractiveness. Furthermore, our behavioral experiments indicated that this species uses floral scent to attract the honey bee while discouraging the ineffective hornet and bumblebee. Honey bees in the Y-tubes were drawn more to the flowers than to the leaves, supporting a role for floral scent in pollinator attraction^12^,^13^. In contrast, the hornets and bumble bees were repelled by both types of tissue. This was further evidence for a dual role in attraction/repulsion, with nectar acting as an ecological filter.

Plants respond to insect feeding damage by releasing varying kinds and levels of volatiles at the site of the injury^17^. These emissions are one of the most important and immediate responses to herbivory^19^,^20^,^33^,^34^. For example, the monoterpene volatiles of *Chrysanthemum morifolium* repel ovipositing females of the diamondback moth *Plutella xylostella*^35^. Our detected compounds also seemed to defend the flowers and leaves against attack by beetles. Damage to those tissues stimulated changes in relative compositions and increased the release of Elsholtzia ketone, making our plants less attractive to those herbivores.

Over time, certain volatile compounds from *E*. *rugulosa* plants have been selected instead of others by pollinators. First, Asian honey bees demonstrate a highly significant preference for flowers, perhaps because they show an innate tendency toward stronger odors. Second, these bees possibly learn to associate a distinctive floral scent with its presence in newly opened flowers, so that the odor effectively acts as an honest signal that increases pollination efficiency. Third, a particular scent might function as a warning signal, thus reducing the likelihood of visits by hornets and bumble bees, and keeping flowers from becoming damaged. Those volatiles might function as good markers of *E*. *rugulosa* identity, thus discouraging visits from beetles. In addition, the herbivore-induced volatiles may serve as reliable signals to other herbivores regarding the presence of competitors or danger from herbivore enemies. Finally, it could be true that volatiles have direct toxic action on herbivores. Such possibilities could be further tested under biologically realistic conditions and with appropriate concentrations of chemical compounds.

In conclusion, our data demonstrate that volatile compounds produced by *E*. *rugulosa* function as an attractant for legitimate pollinators but deter ineffective pollinators while also defending against insect herbivores. When compared with other plant species, we believe that the roles played by scents in *E*. *rugulosa* are the most diverse. Therefore, this species provides a striking example of multiple, combined functions that result from selection pressure due to both biotic and abiotic factors. Future comparative studies of volatile compounds from species closely related to *E. rugulosa* and the behavioral response of insects to these compounds may be particularly useful in improving our understanding of the evolution and ecological significance of scents in plant–animal interactions.

## Methods

### Study species

*Elsholtzia rugulosa* is an herb to subshrub with a height of 30 to 150 cm and grows at elevations of 1300 to 2800 m in southwestern China. This important nectar plant flowers between October and December. In November 2014, we sampled a natural population of approximately 400 flowering plants in Ailaoshan, Jingdong, western Yunnan Province, SW China (N 25°02′, E 99°51′).

### Behavioral experiments

To test the importance of floral scent as an attractant, we performed insect behavior experiments in the laboratory with a glass Y-tube (20 mm in diam.; each branch, 90 mm long; main tube, 170 mm long)^12^,^13^. The Y-tube was placed on a light table, and the room was otherwise kept dark by covering windows with black curtains. The main arm was connected to a suction pump, such that air was drawn along every arm of the Y-tube. After each arm was connected to a Nalophan bag or a glass container containing an odor source, a hole was made in each to allow airflow. *Apis cerana* (*n* = 37), occasional visitors *Vespa velutina* (*n* = 18) and *Bombus* sp. (*n* = 15), and beetles (*n* = 20) were captured and kept in individual containers (20 cm^2^) for up to 20 min prior to the start of the experiments. The insects were placed at the entrance and allowed to walk down the Y-tube to select one of the branches.

### Collection and analysis of volatile compounds

Scents from intact and damaged inflorescences, and from normal and damaged leaves, were collected outdoors by methods utilizing dynamic headspace adsorption^36^. The newly opened inflorescences were enclosed in a polyethylene terepthalate cooking bag (25 × 38 cm). Two holes were cut at opposite ends – one hole was fitted with an activated carbon filter for air intake, the other with a Super-Q volatile collection trap (Analytical Research Systems) that contained 30 mg of Alltech Super-Q adsorbent material. Each flower was enclosed for 2 h, after which its headspace was sampled with two micropumps driven by a portable battery. Airflow was adjusted to a constant 100 mL min^−1^ with a flow meter. Sampling periods were 3 to 4 h long. Empty cooking bags placed in close proximity to the inflorescences were used as controls. After the fragrance sampling was completed, adsorbed volatiles were eluted from the Super-Q with 1.5 mL of dichloromethane (Uvasol; Merck), then sealed in glass vials and stored at –20 °C. Scent samples were collected during the sunniest time of the day, between 12:00 and 16:00, which coincided with when the floral visitors normally are present. Leaves were damaged in a manner that mimicked beetle attack in the field^37^, i.e., puncturing five to six holes in each leaf with a puncher. Inflorescences were also damaged on six to eight detached flowers.

The volatiles were analyzed on a Hewlett-Packard 6890 Series GC System coupled to a Hewlett-Packard 5973 Mass Selective Detector and an Agilent 7683 Series Automatic Liquid Sampler. An HP-5MS column (5% phenylmethylpolysiloxane; 60 m long, 0.32 mm inner diam., 0.25 μm film thickness) was used. Constant helium gas flow was controlled electronically at 1.4 mL min^−1^. The GC oven temperature began at 50 °C and was increased by 5 °C min^−1^ to 100 °C, held for 10 min, then increased by 5 °C min^−1^ to 280 °C and held for 5 min. The MS interface was 280 °C and the ion trap was activated at 150 °C. Mass spectra were taken at 70 eV (in EI mode) with a scanning speed of 1 per scan from m/z 35 to 550. All compounds were compared against the Wiley NIST 05 mass spectral database and Wiley 7.

### Statistical analysis

The results from each behavioural experiment were compared using a binomial test to establish if wasps showed overall preference. All analyses were performed using SPSS 16 (SPSS Company, Chicago, Illinois, USA), with measured variables presented as mean ± SE.

## Additional Information

**How to cite this article**: Zhang, F.-P. *et al*. Multiple functions of volatiles in flowers and leaves of *Elsholtzia rugulosa* (Lamiaceae) from southwestern China. *Sci. Rep*. **6**, 27616; doi: 10.1038/srep27616 (2016).

## Figures and Tables

**Table 1:** Occurrence and relative amounts of volatile compounds emitted by *Elsholtzia rugulosa* flowers and leaves.

Compound	Retention time	Relative amount (%)			
		Flower (*n* = 3)	Damaged flower (*n* = 3)	Leaf (*n* = 5)	Damaged leaf (*n* = 4)
Elsholtzia ketone	20.08	9.98 ± 1.26	25.87 ± 5.93	24.76 ± 4.96	79.35 ± 8.07
Piperitone	21.72	0.93 ± 0.38	4.79 ± 2.02	1.59 ± 0.37	0.67 ± 0.50
β-Elemene	24.19	0.98 ± 0.29	0.42 ± 0.07	–	–
β-Bourbonene	25.54	3.63 ± 0.48	3.30 ± 0.50	–	0.76 ± 0.08
β-Caryophyllene	26.48	45.89 ± 2.59	40.24 ± 3.42	3.28 ± 1.50	3.77 ± 0.14
α-Caryophyllene	27.37	5.56 ± 0.22	4.12 ± 0.35	–	0.36 ± 0.02
Caryophyllene oxide	30.59	2.16 ± 0.29	3.17 ± 1.74	–	0.82 ± 0.23
